# Mechanistic Elucidation of Yiqi Yangyin Qingre Decoction in Diabetic Nephropathy Therapy via Network Pharmacology and In Vivo Validation

**DOI:** 10.1155/jdr/9933955

**Published:** 2026-08-10

**Authors:** Shujiao Zhang, Yiran Xie, Jingyi Tang, Xi Guo, Yuxin Hu, Yuzi Cai, Lin Wang, Xiaole Guo, Mengqi Zhou, Xu Wang, Yuanyuan Deng, Yu Ma, Yaoxian Wang, Wei Jing Liu

**Affiliations:** ^1^ Dongzhimen Hospital, Beijing University of Chinese Medicine, Beijing, China, bucm.edu.cn; ^2^ Key Laboratory of Chinese Internal Medicine of Ministry of Education, Beijing Dongzhimen Hospital, Beijing University of Chinese Medicine, Beijing, China, bucm.edu.cn; ^3^ Department of Integrated Traditional Chinese and Western Medicine, The First Affiliated Hospital of Zhengzhou University, Zhengzhou, Henan, China, zzu.edu.cn; ^4^ Department of Traditional Chinese Medicine, Beijing Puren Hospital, Beijing, China; ^5^ Department of Nephrology, China–Japan Friendship Hospital, Beijing, China, zryhyy.com.cn; ^6^ Dongfang Hospital, Beijing University of Chinese Medicine, Beijing, China, bucm.edu.cn; ^7^ Henan University of Chinese Medicine, Zhengzhou, China, hactcm.edu.cn; ^8^ Renal Research Institution of Beijing University of Chinese Medicine, Beijing University of Chinese Medicine, Beijing, China, bucm.edu.cn

**Keywords:** autophagy, diabetic nephropathy, lysosomes, pyroptosis, Yiqi Yangyin Qingre decoction

## Abstract

**Background:**

Diabetic nephropathy (DN) is a serious microvascular complication of diabetes that urgently requires effective treatments with low toxicity. The traditional Chinese medicine formula Yiqi Yangyin Qingre decoction (YQYYQR) has demonstrated potential in alleviating DN, yet its pharmacological mechanism remains unclear.

**Methods:**

UPLC‐MS/MS combined with network pharmacology was utilized to qualitatively analyze YQYYQR′s bioactive components and predict therapeutic targets. Integrating public databases and GEO‐derived DN‐related genes, core targets were identified via intersection and subjected to pathway enrichment. Molecular docking validated key component–core target interactions, with in vivo DN mouse experiments and transcriptome sequencing performed for verification.

**Results:**

This study identified 376 bioactive components from YQYYQR, corresponding to 1284 potential therapeutic targets. Cross‐analysis between these targets and DN‐related genes yielded 57 overlapping targets, among which GSK3*β* and NFKB1 were screened as core hub genes. Network pharmacology and transcriptomic pathway enrichment analyses indicated that the mechanism of YQYYQR in intervening DN involves biological processes such as autophagy and inflammatory response. In vivo, YQYYQR significantly improved mouse proteinuria and renal function and alleviated pathological kidney damage. Mechanistically, YQYYQR enhanced the inhibitory phosphorylation of GSK3*β* at Ser9, thereby facilitating TFEB nuclear translocation and activating the autophagy‐lysosomal pathway. Simultaneously, it suppresses the NLRP3/ASC/caspase‐1/GSDMD‐N–mediated pyroptosis pathway, ultimately reducing renal inflammation.

**Conclusion:**

YQYYQR exerts a protective effect against DN progression by targeting core genes including GSK3*β*, regulating the GSK3*β*‐TFEB axis to restore autophagic flux via the autophagy‐lysosomal pathway, and inhibiting NLRP3‐mediated pyroptosis to mitigate excessive renal inflammation.

## 1. Background

With 148.0 million adults living with diabetes—the highest global burden—China faces an imposing and growing diabetic nephropathy (DN) crisis [1]. As a prevalent microvascular complication and a leading cause of end‐stage renal disease, DN poses a substantial clinical challenge [2]. Although current management primarily relies on drugs such as dapagliflozin and valsartan to control hyperglycemia and hypertension, their efficacy is often compromised by adverse effects [3]. This pressing unmet medical need underscores the imperative to discover novel therapeutic strategies that can disrupt DN progression by targeting its underlying mechanisms.

In the pathogenesis of DN, the bidirectional interplay between sustained inflammation and dysregulated autophagy is a critical driver of disease advancement [4, 5]. Autophagy is orchestrated by a sophisticated regulatory network, with transcription factor EB (TFEB) serving as a principal transcription factor governing both autophagy and lysosomal function [6–8]. The subcellular localization and activity of TFEB can be induced by the phosphorylation‐mediated inactivation of glycogen synthase kinase‐3*β* (GSK3*β*), suggesting that activation of the GSK3*β*‐TFEB pathway may counteract autophagic and lysosomal dysfunction in DN [9]. Therefore, therapeutic targeting of this axis holds significant promise for DN treatment.

Accumulating evidence suggests that traditional Chinese medicine (TCM) holds promise for delaying DN progression by multitarget mechanisms and a favorable safety profile [10]. In TCM theory, DN falls under the categories of “edema” and “kidney wasting,” with “deficiency of Qi and Yin, and excessive internal heat” identified as its core pathogenesis [11]. This “deficiency of vital Qi” creates a predisposition to DN, whereas “predominance of pathogenic Qi,” often manifested as chronic inflammation in modern terms, drives disease progression. Guided by this principle, our team developed Yiqi Yangyin Qingre decoction (YQYYQR) to tonify Qi and Yin while clearing heat. Our prior studies have substantiated YQYYQR′s efficacy in ameliorating proteinuria and renal dysfunction in DN models [12]. However, the precise mechanisms underlying its multitarget actions warrant further systematic investigation.

This study elucidates the mechanism of YQYYQR in DN through an integrated strategy of network pharmacology, bioinformatics, and in vivo validation. Our findings bridge TCM′s holistic principles with modern precision medicine, illustrating a novel interplay between autophagy, inflammation, and pyroptosis in DN pathogenesis. This work establishes a scientific foundation for the clinical application of YQYYQR and informs future personalized therapeutic strategies for DN.

## 2. Methods

### 2.1. Preparation of YQYYQR

YQYYQR was composed of eight Chinese herbs, with 50 g of Radix astragali as the principal herb, complemented by 2 g of Cordyceps cicadae, 15 g of Radix rehmanniae, 15 g of *Ligustrum lucidum*, 15 g of Radix scrophulariae, 15 g of Radix Scutellariae, 15 g of *Forsythia suspensa*, and 18 g of Arctium fruit. The medicinal materials used in this decoction were provided by the Beijing University of Chinese Medicine. The composition details in YQYYQR (one dose) are shown in Table 1. After boiling and concentrating all the herbs, the resulting extract was designated as the medium dose (YQYYQR‐M) and stored in a −80°C refrigerator for subsequent use.

**Table 1:** The composition of YQYYQR.

Pharmaceutical name	Botanical plant or animal name	Parts used	Chinese name	Amount (g)
Radix astragali	*Astragalus membranaceus* (Fisch.) Bunge	Root	Huangqi	50
Cordyceps cicadae	Isaria cicadae Miquel	Fruit	Chanhua	2
Radix rehmanniae	*Rehmannia glutinosa* (Gaertn.) Libosch. ex Fisch. and C. A. Mey.	Root	Shengdihuang	15
*Ligustrum lucidum*	*Ligustrum lucidum* Ait.	Fruit	Nvzhenzi	15
Radix scrophulariae	*Scrophularia ningpoensis* Hemsl.	Root	Xuanshen	15
Radix Scutellariae	*Scutellaria baicalensis* Georgi	Root	Huangqin	15
*Forsythia suspensa*	*Forsythia suspensa* (Thunb.) Vahl	Fruit	Lianqiao	15
Arctium fruit	*Arctium lappa* L.	Fruit	Niubangzi	18

### 2.2. Ultrahigh Performance Liquid Chromatography Coupled to Tandem Mass Spectrometry (UPLC‐MS/MS) Analysis of YQYYQR Aqueous Extracts

The sample of TCM medicine solution was thawed on ice, vortexed for 10 s, and mixed thoroughly. A 200‐*μ*L sample was placed in a 1.5‐mL centrifuge tube and mixed with the extraction solution consisting of 20% acetonitrile and methanol (1:1, v/v) containing the internal standard. Following vortex centrifugation, a 350‐*μ*L portion of the supernatant was collected and concentrated to complete dryness at 20°C. Subsequently, 150 *μ*L of 70% methanol extract was added for reconstitution. The sample was vortexed for 3 min, sonicated in an ice water bath for 10 min, and centrifuged at 12,000 r/min at 4°C for 3 min. Finally, a 120‐*μ*L aliquot of the supernatant was loaded into the corresponding lining pipe for machine analysis.

The multicomponent fingerprint spectrum of YQYYQR was characterized by UPLC‐MS/MS using a Vanquish system (Thermo Fisher Scientific, United States). Chromatographic analysis was achieved using an ACQUITY BEH C18 column (100 × 2.1 mm, 1.8 *μ*m), with detection at 280 nm. The linear gradient is listed in Table 2, with a flow rate of 0.35 mL/min and an injection volume of 2 *μ*L. Furthermore, mass spectrometry was conducted using the Thermo Q EXACTIVE system.

**Table 2:** Mobile phase conditions of chromatographic separation.

Times (min)	Flow rate	A (%) water	B (%) acetonitrile
0	500	85	15
11	500	25	75
12	500	2	98
14	500	2	98
14.1	500	85	15
15	500	85	15
16	500	85	15

### 2.3. Prediction of YQYYQR Bioactive Components and Corresponding Targets

The SWISS ADME Online Database (http://swissadme.ch/index.php) was employed to screen the constituents of YQYYQR, identified through UPLC‐MS/MS. The bioactive components of YQYYQR were selected based on criteria specifying that gastrointestinal absorption (GI absorption) was “high,” and at least two of the five predicted properties (Lipinski, Ghose, Veber, Egan, and Muegge) were marked as “YES.” Subsequently, the compound names were uploaded to the PubChem database (https://pubchem.ncbi.nlm.nih.gov/) to retrieve the canonical SMILES structures of the components deemed bioavailable. These structures were then submitted to the SwissTargetPrediction database (http://www.swisstargetpredict.ch/) for target prediction, applying a screening criterion of probability > 0. Finally, the predicted targets were verified and refined using the UniProt database (http://www.uniprot.org/).

### 2.4. Screening of Potential Targets for DN

Target genes associated with DN were retrieved from several open‐source databases, including GeneCards (https://www.genecards.org), OMIM (https://www.omim.org/), DisGeNET database (https://www.disgenet.org), and the DrugBank (https://go.drugbank.com/). Relevant disease targets for DN were identified using the keyword “diabetic nephropathy.” For the GeneCards database, a minimum relevance score of 10 was required for search results, whereas the DisGeNET database required a score of ≥ 0.03 for searching relevant targets. The data obtained from these sources were merged, duplicates were eliminated, and the final list of DN‐related targets was compiled.

### 2.5. Potential Targets of YQYYQR in DN Treatment

Expression profiling data from GSE1009 were obtained from the Gene Expression Omnibus (GEO) database (http://www.ncbi.nlm.nih.gov/geo/) [13]. A total of six samples were analyzed in this dataset, consisting of three normal human kidney controls and three samples from patients with DN. Gene identification was performed using the probe ID, as facilitated by the Bioconductor package in the R software. Differentially Expressed Genes (DEGs) were initially detected using a two‐tailed *t*‐test with Benjamini–Hochberg correction, followed by limma‐based screening between DN and control samples (*p* < 0.05, ∣log2FC | >1). Volcano plots and clustered heatmaps were created with ggplot2 to depict expression patterns. The resulting intersecting targets were recognized as the central molecular targets of YQYYQR, and further analyses were conducted in the context of DN.

The intersecting targets among YQYYQR candidate compounds, GSE1009 transcriptomic data, and disease‐associated targets were systematically identified and visualized through comparative analysis using the Venn diagram platform (http://bioinformatics.psb.ugent.be/webtools/Venn/). These overlapping targets represent the core therapeutic targets of YQYYQR for combating DN, forming the foundation for subsequent mechanistic investigations.

### 2.6. Gene Function and Pathway Enrichment

Gene Set Enrichment Analysis (GSEA) was employed to assess whether a predefined set of genes exhibited statistically significant differences in consistency between two biological states. Following 1000 simulated calculations of the expression spectral matrix, the enrichment results were obtained. Gene Ontology (GO) functional analysis and Kyoto Encyclopedia of Genes and Genomes (KEGG) pathway analysis were conducted through the DAVID database (http://david.abcc.ncifcrf.gov), with subsequent visualization executed using R packages. *p* values < 0.05 were used to identify significant results for further analysis. The 10 most significantly enriched terms from both GO and KEGG analyses were selected, ensuring a focused presentation of paramount biological pathways and functional classifications.

### 2.7. Protein–Protein Interaction (PPI) Network

Intersecting YQYYQR‐DN targets were analyzed in STRING (https://string-db.org/), limited to human proteins with a confidence cutoff of 0.400. The network was subsequently visualized and interpreted in Cytoscape (3.8.2). The Cytohubba plugin, integrated within Cytoscape, was employed to efficiently identify hub genes. The top 10 hub genes were determined according to the maximum neighborhood component (MCC) and degree centrality.

### 2.8. Molecular Docking

Molecular docking validation was performed between the hub genes and YQYYQR, as well as the primary bioactive components selected from the “component–target–pathway” analysis. Initially, the 2D and 3D structures of the ligands, including moupinamide (PubChem CID: 5280537), cis‐3‐hexenol glucoside (PubChem CID: 5318045), rutamarin (PubChem CID: 26948), wogonin (PubChem CID: 5281703), and palmatine (PubChem CID: 19009), were downloaded from the PubChem database (https://pubchem.ncbi.nlm.nih.gov/). The 2D and 3D crystal structures of the core target proteins were retrieved from the Protein Data Bank (PDB) (http://www.rcsb.org/). These structures were then converted into Mol2 format files using Open Babel 2.4.1 software, imported into AutoDockTools 1.5.7 software. Hydrogenation treatment of the proteins, hydrogenation of small molecules, and determination of reversible bonds were performed with AutoDockTools 1.5.7. The binding pocket coordinates and grid box dimensions for each target protein were set according to the active site information (Table 3). Subsequently, key active ingredients were docked with target proteins via AutoDock Vina 1.2.0, yielding the docking binding energy and the corresponding docking result file. Finally, docking conformations were visualized using Discovery Studio Visualizer 2019 and PyMOL 2.3.0 software [14].

**Table 3:** Hub gene protein pocket coordinates and Grid box sizes.

Target gene	PDB ID	Protein pocket coordinates			Grid box size
		*X*	*Y*	*Z*	
HSP90AA1	4BQG	−17.837	−12.310	10.720	25 × 28 × 28
NFKB1	1SVC	42.464	14.683	38.036	50 × 50 × 50
GSK3B	1J1B	22.000	15.000	−12.000	23 × 31 × 21
EZH2	5WG6	83.26	4.00	−54.92	24 × 24 × 24
EP300	8QZC	17.837	12.310	10.720	80 × 68 × 76
MCL1	6OQD	17.280	4.280	16.260	20 × 20 × 20
STAT1	1SVC	26.001	10.079	142.606	47.25 × 23.25 × 23.25
HNF4A	3FS1	82.95	98.22	98.22	50 × 50 × 50

### 2.9. Animals and Experimental Procedures

Specific pathogen‐free (SPF)–grade male db/db mice (8 weeks old) and age‐matched db/m controls were acquired from Beijing Vital River Laboratory Animal Technology Co. Ltd. All procedures in this study were performed following internationally recognized principles of laboratory animal care and use, and the experimental protocol was approved by the Animal Protection and Ethics Committee of Dongzhimen Hospital, Beijing University of Chinese Medicine (BUCM‐2023042003‐2110).

All animals were housed in SPF‐grade rooms with adequate ventilation, constant temperature (22^°^C ± 2^°^C), relative humidity (50*%* ± 10*%*), and a 12‐h light/dark cycle, with unrestricted access to food and water. Following a 2‐week adaptation period, tail‐vein glucose was determined, and mice presenting random levels ≥ 16.7 mmol/L (300 mg/dL) for three consecutive days were identified as DM [15]. Subsequently, the mice were randomly assigned to the DN, dapagliflozin, YQYYQR‐H, YQYYQR‐M, and YQYYQR‐L (*n* = 5 each), with db/m mice (*n* = 5) serving as normal controls (NC). Throughout the experimental period, mice were provided with standard feed. The YQYYQR‐L, ‐M, and ‐H groups received oral gavage with YQYYQR at 14.91, 29.81, and 59.62 g/kg/d for 12 consecutive weeks, whereas the NC and DN groups received vehicle (double‐distilled water). The medium dose of YQYYQR (29.81 g/kg/d) was calculated according to the clinical equivalent dose and the body surface area (BSA) normalization method from human to animal [16], and has been validated as an effective therapeutic dose in previous studies [17]. Based on this evidence, a concentration gradient of 0.5:1:2 was established with reference to related literature [18] to further observe the effect of concentration variation on the therapeutic outcomes. Before terminal tissue collection, mice were deeply anesthetized with isoflurane inhalation (3% for induction and 1.5% for maintenance). Blood samples were collected by orbital enucleation under deep anesthesia and centrifuged to obtain serum for biochemical evaluation. Immediately after blood collection, mice were euthanized by cervical dislocation while still under deep anesthesia, in accordance with the approved institutional animal care and use protocol. The kidneys were harvested for subsequent calculation of the organ‐to‐body weight ratio (kidney index [KI]). No mice showed abnormal signs or reached humane endpoints throughout the experiment.

### 2.10. Enzyme‐Linked Immunosorbent Assay (ELISA)

The urinary albumin (UALB) concentration was detected by a Mouse ALB ELISA Kit (Fine Biotech, Wuhan, China), and creatinine (Cr) concentration was determined with a Creatinine Colorimetric Assay Kit (Elabscience, Wuhan, China). Urinary protein excretion was evaluated by calculating the UALB‐to‐Cr ratio (UACR, mg/g). Serum IL‐18, IL‐1*β*, IL‐6, and TNF‐*α* were assayed using ELISA kits (Elabscience, Wuhan, China) according to the manufacturer′s guidelines.

### 2.11. Measurement of Biochemical Indicators

After centrifugation of blood samples at 3000 rpm for 10 min, serum was harvested for biochemical testing. Blood urea nitrogen (BUN) concentrations were determined using a urease‐based Urea Assay Kit, whereas serum creatinine (Scr) levels were analyzed by a Creatinine Assay Kit. All assay kits were supplied by Nanjing Jiangcheng Bioengineering Institute.

### 2.12. Histological Analysis

After harvesting, kidney samples were fixed in 4% paraformaldehyde and embedded in paraffin. Tissue sections (3 *μ*m) were prepared and stained with hematoxylin and eosin (H&E), Masson′s trichrome, and periodic acid‐Schiff (PAS). Microscopic imaging was conducted using an Olympus microscope, and semiquantitative evaluation was carried out with ImageJ.

### 2.13. Western Blot

Kidney tissues were lysed in 150 *μ*L RIPA buffer (Applygen, C1053) on ice for 45 min with intermittent shaking. Following centrifugation of lysates at 12,000 rpm for 15 min at 4°C, protein concentrations were determined by BCA assay. Ten micrograms of protein per sample were separated via SDS‐PAGE and transferred to PVDF membranes (Millipore). Membranes were blocked with 5% nonfat milk in TBST for 1 h, then incubated overnight at 4°C with primary antibodies: kidney injury molecule‐1 (KIM‐1) (1:1000, CST, United States), neutrophil gelatinase–associated lipocalin (NGAL) (1:1000, Abcam, United Kingdom), NOD‐like receptor family pyrin domain‐containing 3 (NLRP3) (1:1000, Immunoway, China), apoptosis‐associated speck‐like protein containing a CARD (ASC) (1:500, Santa Cruz, United States), cysteine aspartic acid specific protease (caspase) 1/P20 (1:2000, Proteintech, China), gasdermin D‐N (GSDMD‐N) (1:1000, Abcam, United Kingdom), LC3 (1:2000, Abcam, United Kingdom), P62 (1:10000, Abcam, United Kingdom), GSK3*β* (1:5000, Abcam, United Kingdom), p‐GSK3*β* (Ser9; 1:1000, CST, United States), TFEB (1:2000, ABclonal, China), and *β*‐actin (1:1000, Abcam, United Kingdom). Membranes were washed three times with TBST, incubated with goat anti‐rabbit IgG (1:2000, CST, United States) and goat anti‐mouse IgG (1:2000, CST, United States) for 1 h at room temperature. Signal detection was performed using ECL, and band intensities were measured using ImageJ, normalizing to *β*‐actin.

### 2.14. Immunohistochemical (IHC) Staining

After fixation in cold methanol for 30 min, kidney sections were incubated overnight at 4°C with anti–IL‐6 (1:100, Abcam, United Kingdom), anti–TNF‐*α* (1:100, ABclonal, China), and anti–pSer9‐GSK3*β* (1:50, CST, United States) primary antibodies and subsequently incubated with secondary antibodies. Finally, an HRP‐DAB system (Proteintech, Wuhan, China) and a microscope were used to visualize and image the sections.

### 2.15. Immunofluorescence (IF)

Sections of renal tissue were embedded and prepared into 20‐*μ*m–thick sections. Sections were rewarmed prior to staining, blocked, and then incubated overnight at 4°C with primary antibodies against ASC (1:400, Santa Cruz, United States), caspase 1 (1:200, Proteintech, China), NLRP3 (1:50, Immunoway, China), LC3B (1:400, Abcam, United Kingdom), P62 (1:400, Abcam, United Kingdom), TFEB (1:50, ABclonal, China), lysosomal‐associated membrane protein 1 (LAMP1) (1:200, Abcam, United Kingdom), and cathepsin B (CTSB) (1:200, Proteintech, China). Sections were incubated with fluorescent secondary antibodies in the dark at room temperature, mounted with antifade medium, and observed using a fluorescence microscope.

### 2.16. Transcriptomics

The construction and sequencing of transcriptomic libraries from mouse kidney tissues were performed by Wuhan Servicebio Technology Co. Ltd. The procedure is briefly outlined as follows: Total RNA was extracted from mouse kidney tissues, and sequencing libraries were constructed in strict accordance with the instructions of the specific library preparation kit. To obtain messenger RNA (mRNA), one of the following strategies was adopted: Eukaryotic mRNA was enriched using Oligo(dT) magnetic beads based on its poly(A) tail characteristics, or ribosomal RNA (rRNA) was selectively removed from the total RNA. The enriched mRNA was then fragmented into short pieces using fragmentation buffer. Using these fragments as templates, first‐strand cDNA was synthesized with random hexamer primers, followed by second‐strand cDNA synthesis with the addition of buffer, dNTPs (containing dATP, dCTP, dGTP, and dTTP), and DNA polymerase I. The resulting double‐stranded cDNA was purified using DNA purification beads, followed by end repair, A‐tailing, and ligation of sequencing adapters compatible with the Illumina platform. The fragments were then size‐selected using DNA purification beads, and the cDNA library was finally enriched by PCR amplification. After passing quality control, libraries were pooled proportionally according to the target sequencing depth and subjected to paired‐end sequencing on the Illumina NovaSeq platform using sequencing‐by‐synthesis technology.

Raw image data generated by the sequencer were converted into FASTQ format files via base‐calling software, containing sequence information and corresponding quality scores. Except for differential expression analysis, which used raw count data as input (with software such as DESeq2 or edgeR suitable for count‐based methods), subsequent analyses were performed based on normalized FPKM values. When generating gene expression heatmaps, FPKM values were subjected to unit variance scaling (UV scaling), that is, *Z*‐score transformation, to highlight expression pattern differences of genes across samples.

### 2.17. Statistical Analysis

Statistical analyses were performed with SPSS 20.0 and GraphPad Prism 8.0. Data were expressed as the mean ± standard deviation (SD). Each independent experiment was conducted in triplicate to obtain an average result. Student′s *t*‐test was utilized to analyze the two‐group comparisons, whereas one‐way ANOVA was employed for comparisons across multiple groups. When significant differences were identified, post hoc tests were used for pairwise comparisons. In cases where normality assumptions were not met, the Wilcoxon rank‐sum test was employed. *p* < 0.05 was regarded as significant.

## 3. Results

### 3.1. UPLC‐MS/MS Chromatograms and Network Analysis of YQYYQR

**Figure 1:**
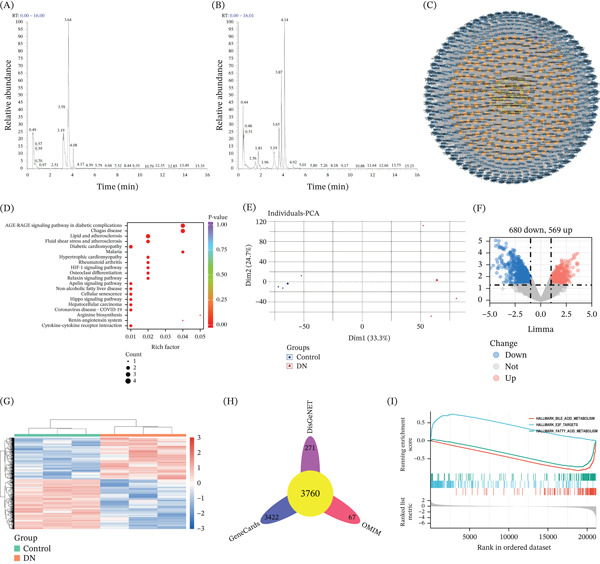
Integrated analysis of YQYYQR composition and DN targets using network pharmacology and bioinformatics. (A) UPLC‐MS/MS chromatograms and network analysis of YQYYQR in negative and (B) positive ion modes. (C) Component–target–pathway–disease network diagram. (D) Bubble chart of KEGG enrichment analysis of targets. (E) PCA diagram. (F) Volcano map of DEGs. (G) Heatmap of DEGs. (H) Number of DN‐related target genes identified across three open‐source databases. (I) GSEA of DEGs.

The multicomponent landscape of YQYYQR was characterized using UPLC‐MS/MS, with representative chromatograms shown in Figure 1A–B. Eight major components were identified (Table 4), and a comprehensive screen of the SWISS ADME database revealed 376 bioactive constituents. After standardization and deduplication in the UniProt database, 1284 unique protein targets were mapped. An herb–compound–target network was constructed to elucidate the interactions, comprising 1530 nodes and 6587 edges (Figure 1C). Topology‐based prioritization highlighted the top 10 bioactive compounds—including wogonoside, arginine, and forsythin—as key mediators of YQYYQR′s effects. Furthermore, KEGG enrichment analysis indicated significant involvement in the AGE‐RAGE signaling pathway in diabetic complications, among other pathways (Figure 1D).

**Table 4:** UPLC‐MS data of YQYYQR.

Number	Ingredients	Chemical formula	Selected ion	Mass‐to‐charge ratio (m/z)	MS2
1	Wogonoside	C_22_H_20_O_11_	[M + H]^+^	461.10702	461.105603
2	Arginine	C_6_H_14_N_4_O_2_	[M + H]^+^	175.11922	175.118961
3	Forsythin	C_27_H_34_O_11_	[M + FA]^−^	579.20768	579.205004
4	Apigenin 7‐O‐glucuronide	C_21_H_18_O_11_	[M−H]^−^	445.07763	269.047335
5	2‐[4‐[(3R,3aR,6S,6aR)‐3‐(3,4‐dimethoxyphenyl)‐1,3,3a,4,6,6a‐hexahydrofuro[3,4‐c]furan‐6‐yl]‐2‐methoxyphenoxy]‐6‐(hydroxymethyl)oxane‐3,4,5‐triol	C_27_H_34_O_11_	[M + NH_4_]^+^	552.24416	355.155034
6	MALIC ACID	C_4_H_6_O_5_	[M−H]^−^	133.01428	133.014055
7	Fisetin	C_15_H_10_O_6_	[M + H]^+^	287.05473	287.055196
8	CAFFEATE	C_9_H_8_O_4_	[M−H_2_O + H]^+^	163.03880	163.0385

### 3.2. Prediction for the Targets of DN

Analysis of the GSE1009 dataset, comprising three healthy control and three DN renal tissue samples (Table 5), revealed a clear separation between DN and normal tissues in principal component analysis (PCA; Figure 1E), confirming the dataset′s reliability. We identified 1249 DEGs, with 569 upregulated and 680 downregulated (Figure 1F). A heatmap of these DEGs showed distinct clustering between disease and normal states (Figure 1G), confirming their representativeness. Integration of 3760 known DN‐associated targets from GeneCards, OMIM, and DisGeNET databases further contextualized the DEGs (Figure 1H). GSEA indicated that the DEGs were significantly enriched in pathways central to DN pathophysiology, including bile acid and fatty acid metabolism, the G2M checkpoint, xenobiotic metabolism, and oxidative phosphorylation (Figure 1I).

**Table 5:** Description of GSE1009 of gene expression profiles for DN.

DN	Ample size (DN/average)	Platform	Number of differential genes	Organism
GSE1009	6 (3/3)	GPL8300 (Affymetrix Human Genome U95 Version 2 Array)	1249	Human

### 3.3. Screening of YQYYQR‐DN Common Target Genes

**Figure 2:**
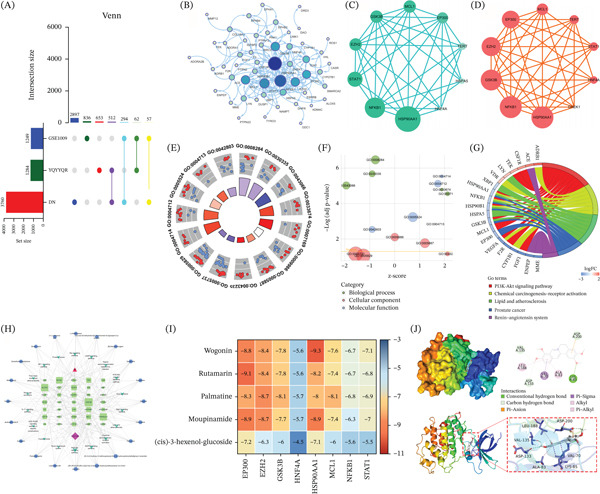
Identification, functional enrichment, and molecular docking validation of key targets of YQYYQR in DN. (A) Venn diagram illustrating common target genes between GEO, DN, and YQYYQR. (B) Protein–protein interaction network. (C–D) The top 10 hub genes identified by two distinct algorithms. (E–F) Top five terms from the GO enrichment analysis. (G) Key pathways and therapeutic targets identified for YQYYQR treatment of DN. (H) Chemical composition–potential target–pathway network diagram of YQYYQR for DN treatment. Green indicates potential gene targets, blue denotes active ingredients of YQYYQR, cyan represents the most significant signaling pathways, and gray lines illustrate their interactions. (I) Heatmap showing the binding energy of interactions. (J) Three‐ and two‐dimensional binding modes of GSK3*β*‐palmatine.

The Venn diagram identified 57 intersection targets between YQYYQR and DN (Figure 2A). A PPI network was constructed from these targets using the STRING database and visualized in Cytoscape (Figure 2B),resulting in a network of 53 nodes and 144 edges after filtering. Hub genes were subsequently identified using the CytoHubba plugin with two topological algorithms (MCC and Degree; Figure 2C–D). The consensus top hub genes, including GSK3β, HSP90AA1, NFKB1, STAT1, EZH2, MCL1, EP300, TERT, and HNF4A, were identified as the core regulatory machinery. These targets are predominantly implicated in cell death and inflammatory response pathways, providing critical insights for elucidating the pathogenesis of DN and revealing its potential therapeutic targets.

### 3.4. Biological Function and Pathway Analysis

To delineate the multifaceted biological functions and mechanisms underpinning YQYYQR′s ameliorative effects on DN, the 57 common target genes were analyzed through GO and KEGG enrichment. As depicted in Figure 2E–F and Table 6, in BP, key intersection targets were primarily enriched in pathways promoting cell proliferation and migration while negatively regulating apoptosis. CC analysis indicated predominant localization to plasma membrane integral components, receptor complexes, and cytoplasm. Moreover, enriched MF terms included protein serine/threonine/tyrosine kinase activity, transmembrane receptor protein tyrosine kinase activity, and ATP binding, among others.

**Table 6:** Supplementary information on GO analysis.

ID	Description
GO:0008284	Positive regulation of cell proliferation
GO:0030335	Positive regulation of cell migration
GO:0043066	Negative regulation of apoptotic process
GO:0033674	Positive regulation of kinase activity
GO:0007169	Transmembrane receptor protein tyrosine kinase signaling pathway
GO:0009986	Cell surface
GO:0005887	Integral component of plasma membrane
GO:0043235	Receptor complex
GO:0005737	Cytoplasm
GO:0005829	Cytosol
GO:0004714	Transmembrane receptor protein tyrosine kinase activity
GO:0004712	Protein serine/threonine/tyrosine kinase activity
GO:0005524	ATP binding
GO:0004713	Protein tyrosine kinase activity
GO:0042803	Protein homodimerization activity

KEGG pathway analysis identified the top five DN‐relevant pathways, including PI3K‐Akt signaling, chemical carcinogenesis‐receptor activation, lipid metabolism, and atherosclerosis, among others (Figure 2G). Notably, several of these pathways are closely associated with autophagy regulation and inflammatory responses. To further elucidate YQYYQR′s mechanism of action, a component‐pathway network was constructed, visualizing the connections between the top 20 KEGG pathways and the active compounds (Figure 2H). Network analysis revealed extensive interconnections among these pathways, suggesting that the bioactive components of YQYYQR likely produce synergistic therapeutic effects against DN through concurrent modulation of autophagy‐related and inflammatory signaling cascades.

### 3.5. Molecular Docking for Key Targets

To evaluate the interactions between core targets and key bioactive compounds, molecular docking was performed. The calculated binding energies were as follows: HSP90AA1‐wogonin (−9.3 kcal·mol^−1^), NFKB1‐palmatine (−6.8 kcal·mol^−1^), GSK3*β*‐palmatine (−8.1 kcal·mol^−1^), EZH2‐palmatine (−8.7 kcal·mol^−1^), EP300‐rutamarin (−9.1 kcal·mol^−1^), MCL1‐palmatine (−8.1 kcal·mol^−1^), STAT1‐wogonin (−7.1 kcal·mol^−1^), and HNF4A‐palmatine (−5.7 kcal·mol^−1^) (Figure 2I). With the exception of the HNF4A‐palmatine complex, all other pairs demonstrated strong binding affinities (binding energy < −7.2 kcal·mol^−1^), suggesting these interactions are functionally relevant. Notably, the high‐affinity binding of palmatine to GSK3*β*, a central hub in autophagy regulation, was further visualized and analyzed using PyMOL and Discovery Studio to elucidate its binding mode (Figure 2J).

### 3.6. YQYYQR Improved the Indicators of Renal Function in DN Mice

**Figure 3:**
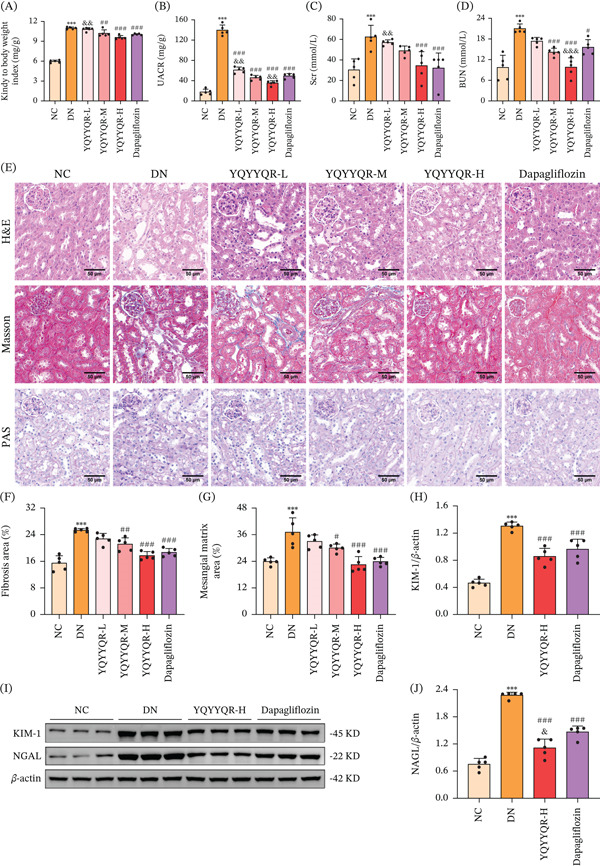
YQYYQR improves renal function and alleviates histopathological damage in DN mice. (A) Kidney‐to‐body weight ratio (*n* = 5), (B) UACR (*n* = 5), (C) Scr levels (*n* = 5), and (D) BUN levels (*n* = 5). (E) Representative histopathological images of kidney sections stained with H&E, Masson′s trichrome, and PAS (*n* = 5; ×400). (F) Fibrosis area. (G) Mesangial matrix area. (H–J) Relative protein expression of KIM‐1 and NGAL (*n* = 5). ^***^ *p* < 0.001 versus NC group. ^#^ *p* < 0.05, ^##^ *p* < 0.01, and ^###^ *p* < 0.001 versus DN group. ^&&^ *p* < 0.01 and ^&&&^ *p* < 0.001 versus dapagliflozin group.

Urinary protein excretion serves as a sensitive indicator of glomerular dysfunction, whereas Scr and BUN are key clinical biomarkers of renal function. As shown in Figure 3A–D, compared with the NC group, the DN group exhibited significantly increased KI (*p* < 0.001; Figure 3A), UACR (*p* < 0.001; Figure 3B), Scr (*p* < 0.01; Figure 3C), and BUN (*p* < 0.001; Figure 3D), indicating marked renal hypertrophy and impaired renal function. Treatment with YQYYQR at low (YQYYQR‐L), medium (YQYYQR‐M), and high (YQYYQR‐H) doses, as well as the positive control dapagliflozin, ameliorated these DN‐associated abnormalities to varying degrees. Compared with the DN group, YQYYQR‐L exhibited limited efficacy, as it exerted no significant effects on KI, Scr, and BUN (*p* > 0.05). However, when the concentration of YQYYQR was increased to the medium dose, marked reductions in KI and BUN were observed compared with the DN group (*p* < 0.01 and *p* < 0.001). Notably, further escalation to the high dose (YQYYQR‐H) resulted in the most pronounced renoprotective effects, as only this dose significantly decreased Scr in addition to further suppressing KI and BUN, thereby revealing a clear dose‐dependent enhancement of efficacy. Moreover, all three doses of YQYYQR significantly reduced the DN‐induced elevation in UACR (*p* < 0.001), highlighting its pronounced efficacy in reducing proteinuria. Importantly, among the YQYYQR‐treated groups, the high dose achieved effects on KI and Scr comparable with those of dapagliflozin, while exerting markedly superior reductions in UACR and BUN than dapagliflozin (*p* < 0.01 and *p* < 0.001), underscoring its prominent renoprotective potential. Collectively, these findings demonstrate that YQYYQR exerts dose‐dependent renoprotective effects in DN mice, with YQYYQR‐H providing the most substantial improvements in both physiological and renal functional outcomes.

### 3.7. YQYYQR Ameliorated Renal Histological Injury in DN Mice

To further elucidate the nephroprotective efficacy of YQYYQR on renal injury, we performed comprehensive histopathological assessments using H&E, Masson′s trichrome, and PAS staining across different doses of YQYYQR (L, M, and H) and dapagliflozin (Figure 3E–G). Renal specimens from DN mice exhibited pronounced vacuolization of tubular epithelial cells, luminal detachment, tubular dilation, inflammatory infiltrates, glomerular hypertrophy, and basement membrane thickening. Remarkably, YQYYQR‐H exerted the most robust amelioration of these histopathological alterations in both glomerular and tubular architectures, with effects comparable with those achieved by dapagliflozin, a clinically established treatment for DN. Moreover, Masson′s trichrome staining of renal tissues revealed that, in the NC group, the glomeruli, tubules, and interstitial structures appeared normal, with only a minimal presence of blue‐stained material. However, the blue‐stained areas were notably expanded in the DN group, indicating the progression of renal fibrosis. Among YQYYQR‐treated groups, YQYYQR‐H most effectively diminished the blue‐stained fibrotic areas, effectively alleviating the extent of renal fibrosis, and the quantitative analysis of the fibrotic area further revealed that the antifibrotic efficacy of YQYYQR‐H was on par with that of dapagliflozin. In addition, PAS staining demonstrated significant mesangial expansion, mesangial matrix proliferation, and thickened glomerular basement membranes in DN mice relative to NC mice, all of which are hallmark features of progressive glomerular injury. The renal protective effect of YQYYQR against DN exhibited a dose‐dependent manner. Specifically, the YQYYQR‐H achieved similar efficacy to the positive control drug, dapagliflozin. Consequently, due to its consistent and superior performance across multiple functional and histological parameters, YQYYQR‐H was selected for further mechanistic investigation.

Furthermore, Western blot analysis was employed to assess the expression levels of renal tubular injury markers, KIM‐1 and NGAL (Figure 3H–J). The results revealed significantly elevated expression of both KIM‐1 and NGAL in the DN group compared with NC (*p* < 0.001), whereas treatment with YQYYQR‐H and dapagliflozin led to a pronounced downregulation of these markers (*p* < 0.001). Notably, the NGAL expression in the YQYYQR‐H group was significantly lower than that in the dapagliflozin group (*p* < 0.05), indicating a potent renal tubular protective effect of YQYYQR‐H. These findings strongly suggest that YQYYQR offers substantial protection against the renal injury associated with DN.

### 3.8. DEGs of Kidney Tissues

**Figure 4:**
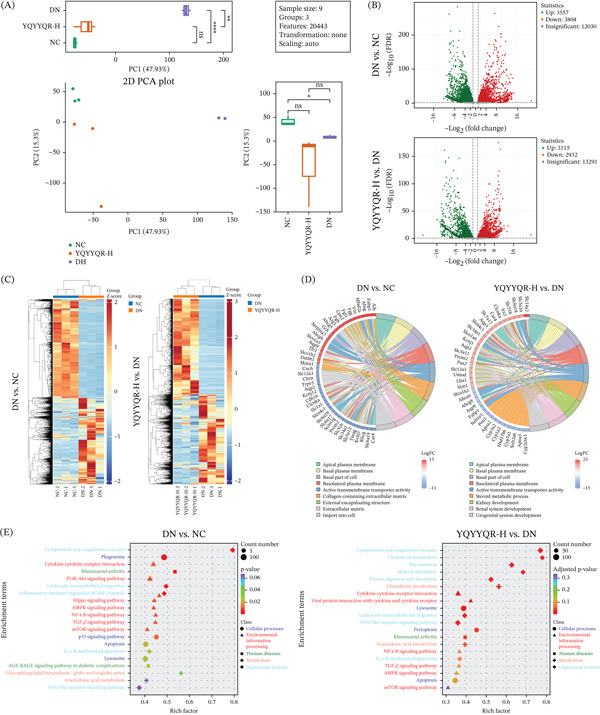
Transcriptomic analysis results. (A) PCA plot of DN, NC, and YQYYQR‐H groups. (B) Volcano plots of DEGs of DN versus NC and YQYYQR‐H versus DN; (C) Cluster heatmap of DEGs of DN versus NC and YQYYQR‐H versus DN; (D) GO annotation of DEGs of DN versus NC and YQYYQR‐H versus DN; (E) KEGG analysis of DEGs of DN versus NC and YQYYQR‐H versus DN.

Transcriptomic analysis of renal tissues revealed significant differences in gene expression patterns among the NC, DN, and YQYYQR‐H groups (Figure 4A). PCA demonstrated a clear separation of the three groups at the transcriptomic level, indicating that both disease induction and herbal intervention significantly influenced the overall gene expression profile of the kidneys. DEGs analysis showed that compared with the NC group, a total of 7361 DEGs were identified in the DN group, of which 3557 genes were upregulated and 3804 were downregulated (Figure 4B). Following intervention with YQYYQR‐H, 6047 genes exhibited significant expression changes compared with the DN group, including 3115 upregulated and 2932 downregulated genes, suggesting that this formula can broadly reverse transcriptomic disturbances under disease conditions (Figure 4B). Hierarchical clustering analysis further confirmed that each group possessed distinct gene expression characteristics (Figure 4C). Functional enrichment analysis of the DEGs revealed that the DEGs between the DN and NC groups were significantly enriched in biological processes such as inflammatory response and autophagy. Notably, when comparing the YQYYQR‐H intervention group with the DN group, its DEGs also showed significant enrichment in related functional terms, including inflammatory response and autophagy process (Figure 4D). KEGG pathway analysis further indicated that the DEGs obtained from both comparative sets (DN vs. NC and YQYYQR‐H vs. DN) were commonly enriched in pathways closely associated with inflammation and autophagy regulation, such as the NOD‐like receptor signaling pathway, NF‐*κ*B signaling pathway, and mTOR signaling pathway. In summary, the transcriptomic data indicate that YQYYQR intervention can induce a widespread reversal of disease‐related gene expression changes (Figure 4E). This multipathway, multitarget regulatory characteristic reflects the “polypotency” of traditional Chinese herbal compound formulations. The convergent enrichment of DEGs from both the disease and treatment groups on specific inflammatory and autophagy pathways provides clear data support for subsequent in‐depth exploration of the formula′s pharmacodynamic material basis and mechanisms of action.

### 3.9. YQYYQR Regulated the Renal GSK3*β*‐TFEB Signaling Axis in DN Mice

**Figure 5:**
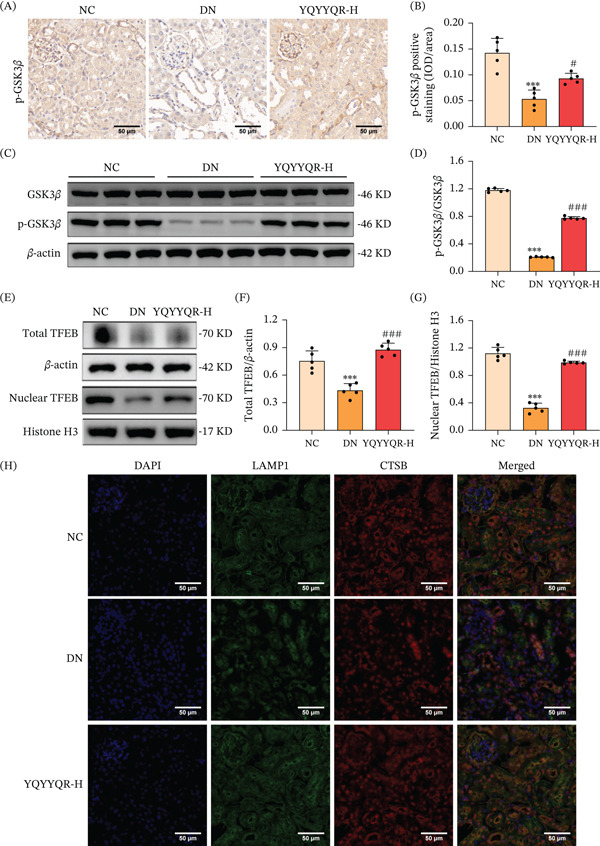
YQYYQR regulated the renal GSK3*β*‐TFEB signaling axis in DN mice. (A–B) The expression level of pSer9‐GSK3*β* in kidney tissue was detected by immunohistochemistry (*n* = 5; ×400). (C–G) Western blot analyses of GSK3*β*, pSer9‐GSK3*β*, total TFEB, and nuclear TFEB expression in kidney tissue (*n* = 5). (H) Immunofluorescence staining of LAMP1 and CTSB (*n* = 5; ×400). ^***^ *p* < 0.001 versus NC group. ^#^ *p* < 0.05, ^###^ *p* < 0.001 versus DN group.

PPI network analysis identified GSK3*β* as a key node with a high degree of value. Accordingly, we explored the role of this potential hub target in mediating the therapeutic effects of YQYYQR‐H by employing IHC and Western blot analysis (Figure 5A–D). IHC analysis revealed that the inactive form phosphorylated GSK3*β* expression at Ser9 (pSer9‐GSK3*β*) was markedly reduced in the kidneys of DN mice compared with the NC group. Notably, administration of YQYYQR‐H effectively restored pSer9‐GSK3*β* levels (Figure 5A–B). As anticipated, Western blot analysis corroborated these findings, demonstrating a significant decrease in the phosphorylation ratio (pSer9‐GSK3*β*/GSK3*β*) in DN mice, which was substantially elevated following YQYYQR‐H treatment (Figure 5C–D), in alignment with the IHC results. Moreover, we assessed TFEB protein expression in renal tissues across different groups via Western blotting (Figure 5E–G). The results revealed that both total and nuclear TFEB protein levels were markedly diminished in the DN model group compared with controls. However, YQYYQR‐H treatment notably restored these levels, indicating that YQYYQR‐induced GSK3*β* inactivation facilitates TFEB nuclear translocation.

To further assess lysosomal stability, we performed double IF staining for LAMP1 and CTSB. LAMP1, a lysosomal membrane marker, appears as green puncta, whereas CTSB, predominantly localized within lysosomes, is visualized as red puncta. As illustrated in Figure 5H, in the NC group, LAMP1 and CTSB exhibited substantial colocalization, and CTSB granules were regularly distributed in a perinuclear pattern, indicating intact lysosomal membranes and preserved function. In contrast, the DN group showed markedly reduced expression of both LAMP1 and CTSB, along with disorganized, diffuse staining and diminished colocalization, suggestive of decreased lysosomal number, impaired proteolytic activity, and lysosomal membrane permeabilization. Notably, these alterations were considerably ameliorated following YQYYQR‐H treatment, indicating its protective effect on lysosomal membrane stability.

Collectively, these data indicate that YQYYQR significantly inactivates GSK3*β* in DN mice, as evidenced by the enhanced expression of inactive pSer9‐GSK3*β*. The consequent inactivation of GSK3*β* further promotes the nuclear translocation of TFEB, thereby facilitating the restoration of lysosomal function.

### 3.10. YQYYQR Unblocked the Autophagy Pathway in DN Mice

**Figure 6:**
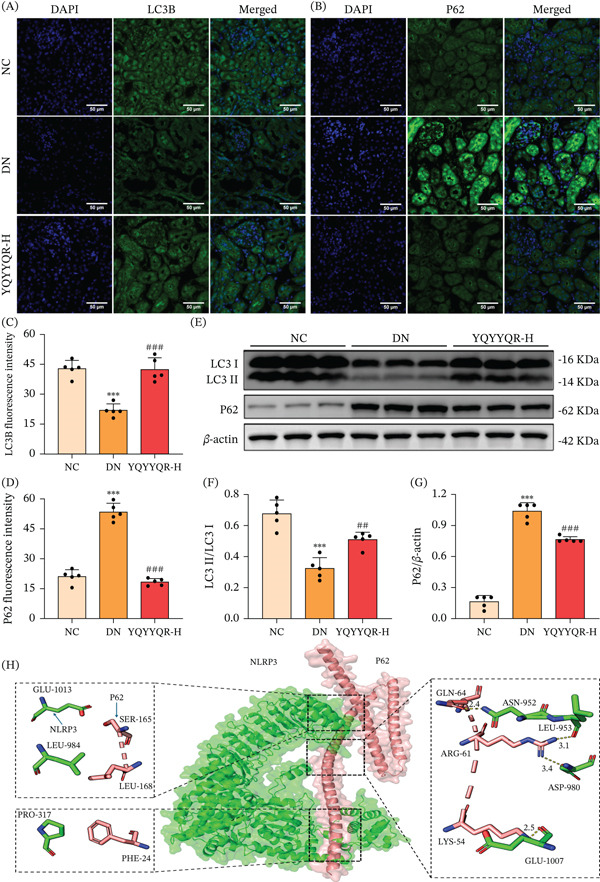
YQYYQR unblocked the autophagy pathway in DN mice. (A, C) Immunofluorescence observation of the expression level of LC3B and (B, D) P62 (*n* = 5; ×400). (E–G) Western blot analyses of LC3 and P62 expression in kidney tissue (*n* = 5). (H) Molecular docking diagram of NLRP3 to P62. ^***^ *p* < 0.001 versus NC group. ^##^ *p* < 0.01, ^###^ *p* < 0.001 versus DN group.

In light of the pivotal role of autophagy in the pathogenesis of DN, we assessed the impact of YQYYQR‐H on autophagosome formation and the expression of autophagy‐related proteins using IF staining (Figure 6A–D) and Western blot analyses (Figure 6E–G). In the DN group, the fluorescence intensity of LC3B and the LC3‐II/LC3‐I ratio (a critical indicator of autophagic flux) were markedly reduced in renal tissues compared with those of the NC group. Conversely, the fluorescence intensity and protein expression of the autophagy receptor protein P62 were significantly elevated. Notably, YQYYQR‐H treatment enhanced the fluorescence intensity of LC3B and the conversion of LC3‐I to LC3‐II, accompanied by a concurrent decrease in P62 expression. These findings suggest that YQYYQR exerts its renoprotective effects in DN by unblocking the autophagy pathway and reducing the accumulation of autophagy substrate P62 within renal tissues.

To further explore the interaction between P62 and key pyroptosis pathway protein NLRP3, we simulated their binding through molecular docking (Figure 6H). It can be seen that there were hydrogen bonding interactions between glu‐1013 and ser‐165, gln‐64 and asn‐952, GLU1007 and LYS54, indicating that P62 interacts with NLRP3.

### 3.11. YQYYQR Mitigated Renal Cell Pyroptosis in DN Mice

**Figure 7:**
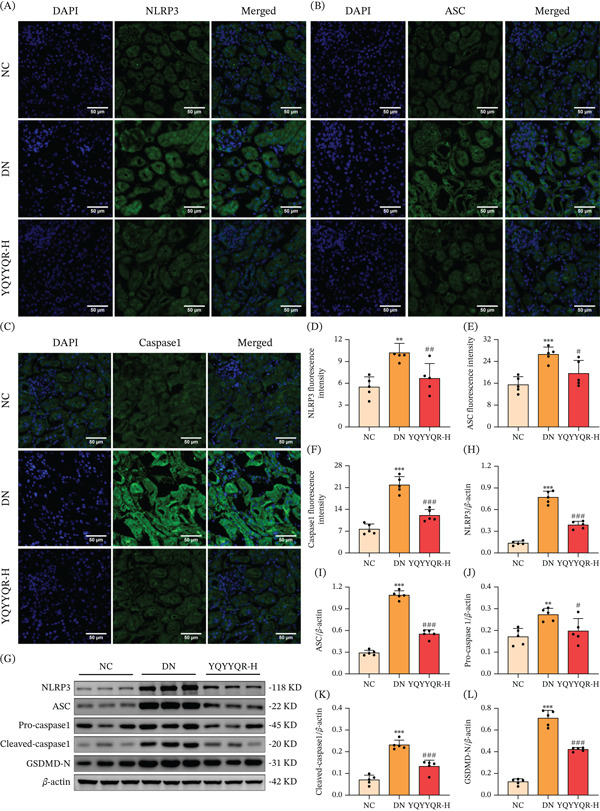
YQYYQR mitigated renal cell pyroptosis in DN mice. Immunofluorescence observation of the expression level of (A, D) NLRP3, (B, E) ASC, and (C, F) caspase 1 protein (*n* = 5; ×400). (G–L) Western blot assay of NLRP3, ASC, procaspase 1, cleaved‐caspase 1, and GSDMD‐N expression in kidney tissue (*n* = 5).^**^ *p* < 0.01, ^***^ *p* < 0.001 versus NC group. ^#^ *p* < 0.05, ^##^ *p* < 0.01, ^###^ *p* < 0.001 versus DN group.

Given the interaction between P62 and NLRP3, we further investigated the expression of pyroptosis‐associated markers to elucidate the regulatory effect of YQYYQR‐H on renal cell pyroptosis in DN mice. IF staining results revealed markedly elevated expression of NLRP3 (Figure 7A,D), ASC (Figure 7B,E), and caspase‐1 (Figure 7C,F) in DN mice relative to the NC. These findings suggest that kidney injury in DN is closely associated with heightened inflammation and pyroptotic cell death. Notably, treatment with YQYYQR‐H significantly attenuated the expression of these pyroptosis markers. Consistent with these observations, Western blot analysis of renal tissues confirmed a similar downregulation of NLRP3, ASC, procaspase 1, cleaved‐caspase 1, and GSDMD‐N protein levels following YQYYQR‐H administration (*p* < 0.05, *p* < 0.01, and *p* < 0.001; Figure 7G–L). Collectively, these results indicate that YQYYQR mitigates renal cell pyroptosis by suppressing activation of the NLRP3/ASC/caspase‐1/GSDMD‐N signaling axis in the kidneys of DN mice.

### 3.12. YQYYQR Repressed Renal Inflammation in DN Mice

**Figure 8:**
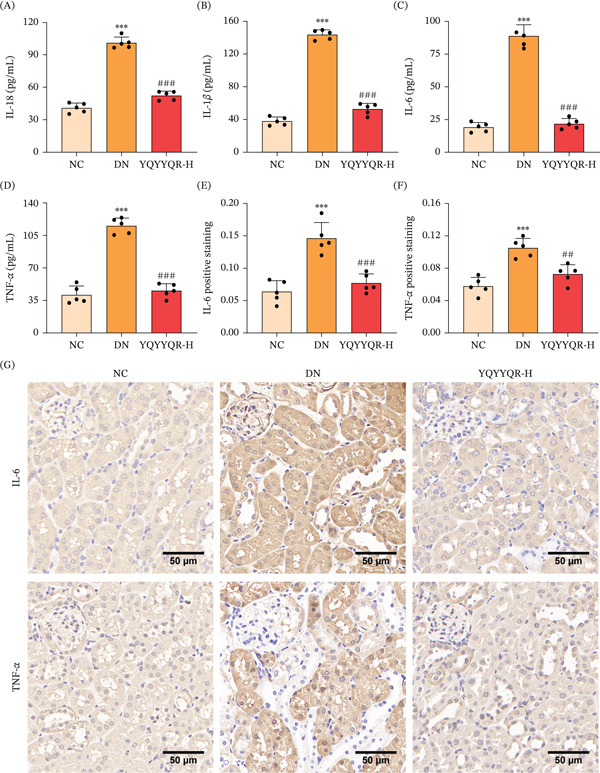
YQYYQR repressed renal inflammation in DN mice. ELISA analysis indicated (A) IL‐18, (B) IL‐1*β*, (C) IL‐6, and (D) TNF‐*α* expression levels in kidney tissue (*n* = 5). (E–G) The expression level of IL‐6 and TNF‐*α* in kidney tissue was detected by immunohistochemistry (*n* = 5; ×400). ^***^ *p* < 0.001 versus NC group. ^###^ *p* < 0.001 versus DN group.

In the pyroptotic cascade, caspase‐1 cleaves pro–IL‐1*β* and pro–IL‐18 to their active forms, triggering the secondary release of IL‐6 and TNF‐*α*, which are key proinflammatory mediators in the pathogenesis of DN. Thus, we investigated whether the renoprotective effects of YQYYQR‐H involve attenuation of this hierarchical inflammatory axis. ELISA analyses first revealed that IL‐18 (Figure 8A) and IL‐1*β* (Figure 8B) levels were dramatically elevated in the DN group (*p* < 0.001), consistent with activated pyroptosis. Concomitantly, downstream IL‐6 (Figure 8C) and TNF‐*α* (Figure 8D) were also markedly increased in the DN group (*p* < 0.001), reflecting a robust proinflammatory state. Moreover, treatment with YQYYQR‐H effectively reversed these elevations, significantly reducing the concentrations of all four cytokines (*p* < 0.001). To further complement these findings, IHC staining revealed that almost no IL‐6 and TNF‐*α* staining was observed in the renal tissue of the NC group. In stark contrast, the DN group demonstrated intense and widespread immunoreactivity for IL‐6 and TNF‐*α*, indicating a pronounced inflammatory response. Notably, treatment with YQYYQR‐H markedly diminished the staining intensity, reflecting a substantial reduction in inflammatory cytokine expression (Figure 8E–G). Collectively, these data demonstrate that YQYYQR mitigates renal inflammation in DN by suppressing a cascade spanning pyroptosis‐driven IL‐18/IL‐1*β* to downstream proinflammatory IL‐6/TNF‐*α*.

## 4. Discussion

In this study, an integrated strategy combining network pharmacology, bioinformatics, and in vivo experiments was employed to systematically investigate the therapeutic mechanisms of YQYYQR in DN. Our results demonstrate that YQYYQR significantly alleviates renal injury in DN mice. This renoprotective effect is likely mediated through multiple mechanisms, including the modulation of inflammatory responses, suppression of pyroptosis, restoration of the autophagy‐lysosome pathway, and regulation of the GSK3*β*‐TFEB signaling.

In the theory of TCM, deficiency of Qi and Yin and excessive internal heat are regarded as the key pathogenesis of DN. The YQYYQR decoction, formulated with eight herbs, was designed to invigorate renal Qi and Yin while clearing heat. Pharmacological studies have substantiated the renoprotective properties of key components contained in the YQYYQR, such as Radix astragali, Cordyceps cicadae, and Radix rehmanniae, which have been shown to reduce proteinuria, improve renal function, and mitigate histopathological renal damage [19]. Our previous studies have demonstrated that YQYYQR ameliorates renal function by suppressing proinflammatory cytokines, modulating the inflammatory response, and restoring autophagic flux [12]. In the present study, network pharmacology analysis identified 57 overlapping targets between YQYYQR and DN, with GSK3*β* emerging as a central hub protein that is intimately linked to autophagy and inflammatory processes, mirroring the complex and multifactorial pathogenesis of DN. Notably, molecular docking revealed strong binding affinities between the bioactive compound palmatine and GSK3*β*, suggesting direct molecular interactions that may underlie the therapeutic efficacy of the formula. This “multicomponent, multitarget” mechanism of action embodies the holistic philosophy of TCM and is consistent with emerging evidence supporting the efficacy of herbal formulations in treating DN through the modulation of autophagy and inflammation‐related pathways [20].

In vivo experiments in db/db mice confirmed the renoprotective effects of YQYYQR, with YQYYQR‐H exhibiting the most pronounced efficacy, as evidenced by marked improvements in renal function and histological outcomes. Mechanistically, we identified GSK3*β* as a potential hub target via PPI network analysis and verified its involvement in YQYYQR‐mediated renal protection. Phosphorylation‐induced inactivation of GSK3*β* has been shown to promote TFEB nuclear translocation [21]. TFEB, the important transcription regulator in the autophagy‐lysosomal pathway, orchestrates lysosomal biogenesis (affecting lysosome number and morphology via genes such as LAMP1), lysosomal enzyme functionality (upregulating hydrolases like CTSB to enhance degradative capacity), and vesicular flux [22]. Consistent with previous literature, YQYYQR was found to enhance the phosphorylation of GSK3*β* at Ser9, thereby inactivating its kinase activity, which in turn promoted the nuclear translocation of TFEB. In addition, CTSB is primarily localized within lysosomes, and its colocalization with the lysosomal membrane marker LAMP1 confirms its intralysosomal distribution [23]. Lysosomal membrane permeabilization can lead to the aberrant leakage of CTSB into the cytoplasm, potentially triggering downstream cytotoxic events [24]. In our study, YQYYQR treatment increased LAMP1‐positive lysosomal structures (indicating restoration of lysosomal morphology/quantity) and elevated CTSB levels (suggesting recovery of degradative function) in DN mice. Concurrently, the improved LAMP1‐CTSB colocalization indicates reduced lysosomal membrane permeabilization and enhanced lysosomal integrity. Our experimental results further demonstrated that YQYYQR upregulated LC3‐II and reduced the accumulation of the autophagic substrate P62. Together with its effects on lysosomal markers, it suggests that YQYYQR may exert its effects by preserving lysosomal integrity and unblocking the autophagy‐lysosome pathway. Furthermore, the blockade of the autophagy pathway was restored, as evidenced by the upregulation of phosphorylated GSK3*β* and the nuclear translocation of TFEB, an autophagy‐lysosome master regulator. These findings are in line with the hypothesis that inhibition of GSK3*β* activity facilitates TFEB nuclear translocation, thereby restoring autophagic homeostasis [25]. Molecular docking and predictive interaction analysis suggested a potential interplay between P62 and NLRP3, raising the possibility that YQYYQR may promote the degradation of NLRP3 by regulating the autophagy‐lysosome pathway, thereby inhibiting inflammasome activation and alleviating inflammation.

Pyroptosis, a proinflammatory cell death, contributes to DN via NLRP3 inflammasome activation [26]. Upon activation, the assembled NLRP3 inflammasome cleaves pro–caspase‐1 to the active form, which then proteolytically cleaves GSDMD‐N to form plasma membrane pores, leading to cell pyroptosis. Simultaneously, caspase‐1 cleaves pro–IL‐1*β* and pro–IL‐18 into their active secreted forms [27]. The release of IL‐1*β* subsequently triggers a cascade involving secondary cytokines IL‐6 and TNF‐*α*, which serve as hallmarks of renal inflammation in DN and actively contribute to glomerulosclerosis and tubulointerstitial fibrosis [28]. Therefore, activation of the NLRP3/ASC/caspase‐1/GSDMD‐N signaling cascade induces inflammatory responses by stimulating cytokine release and recruiting acute inflammatory cells through chemokine‐mediated mechanisms, representing a key upstream regulator of renal inflammation. Inflammation, as a central driver in the pathogenesis of DN, is closely intertwined with disease progression [29]. In our investigation, YQYYQR markedly suppressed the NLRP3/ASC/caspase‐1/GSDMD‐N signaling cascade, while simultaneously mitigating renal inflammation through the downregulation of proinflammatory cytokines. These findings indicate that YQYYQR may suppress pyroptosis pathway activation, thereby limiting inflammatory cytokine release and ultimately alleviating renal inflammatory injury and interstitial fibrosis.

Our study demonstrates that YQYYQR ameliorates DN by modulating the GSK3*β*‐TFEB axis to suppress pyroptosis and restore autophagic‐lysosomal function. This multitarget mechanism is particularly promising, as current frontline therapies (e.g., SGLT2 inhibitors) primarily address metabolic and hemodynamic aspects but do not directly target autophagy‐lysosomal dysfunction or pyroptosis. YQYYQR, with its holistic action, could complement these therapies by addressing these pivotal pathological processes. For instance, a combination of YQYYQR with an SGLT2 inhibitor might synergistically ameliorate hyperglycemia, inhibit inflammatory cell death, and restore cellular clearance mechanisms, offering a more comprehensive strategy to halt DN progression.

However, several limitations should be acknowledged. First, the clinical translation of YQYYQR, as a complex herbal decoction, requires further investigation into its pharmacokinetics, including the bioavailability, tissue distribution, and metabolism of its active constituents. Challenges such as poor solubility or rapid clearance may necessitate advanced formulation strategies to ensure effective renal concentrations. Second, although we identified the GSK3*β*‐TFEB axis as a central pathway, the extensive target list from network pharmacology suggests other mechanisms might contribute to its efficacy. The potential interplay between P62 and NLRP3, for example, warrants deeper exploration to fully elucidate the regulatory network. Third, the experimental sample size was limited. Future studies with larger cohorts are essential to robustly validate its efficacy and better define its therapeutic potential relative to existing treatments.

Our findings underscore that defective autophagy‐lysosomal function and NLRP3‐mediated pyroptosis are critical drivers of DN pathology. We verified that YQYYQR restores autophagic flux, enhances lysosomal integrity, and suppresses the pyroptotic cascade, thereby alleviating renal injury. These effects are mediated, at least in part, through the inactivation of GSK3*β* and subsequent nuclear translocation of TFEB. These insights not only clarify the crucial role of autophagy and pyroptosis in DN but also strongly support further investigation of YQYYQR as a promising multitarget therapeutic candidate.

## 5. Conclusion

Overall, our study provides compelling evidence that the high dose of YQYYQR exerts the most prominent efficacy against DN, which is comparable with that of the positive control. Mechanistically, the renoprotective effects of YQYYQR in DN mice, including attenuation of interstitial fibrosis and repair of renal pathology, may result from its multifaceted actions on the autophagy‐lysosome pathway, pyroptosis suppression, and inflammatory attenuation, potentially mediated by the regulatory GSK3*β*‐TFEB signaling axis. These findings bridge TCM theory with molecular pathophysiology, underscoring YQYYQR as a promising multitarget therapeutic agent for DN and offering a foundation for novel drug development and clinical intervention.

## Author Contributions

Shujiao Zhang: conceptualization, methodology, investigation, resources, conducted experiments, funding acquisition, writing—original draft, formal analysis, writing—review and editing. Yiran Xie: investigation, conducted experiments, writing—original draft, writing—review and editing, formal analysis, software, drew charts, validation, data curation. Jingyi Tang: visualization, data curation. Xi Guo: visualization, data curation. Yuxin Hu: visualization. Yuzi Cai: visualization. Lin Wang: data curation, funding acquisition. Xiaole Guo: data curation. Mengqi Zhou: data curation. Xu Wang: data curation, funding acquisition. Yuanyuan Deng: formal analysis, drew network pharmacology charts, data curation. Yu Ma: formal analysis, drew network pharmacology charts, data curation. Yaoxian Wang: supervision, funding acquisition, project administration. Wei Jing Liu: writing—review and editing, supervision, funding acquisition, project administration. Shujiao Zhang and Yiran Xie have contributed to the work equally and should be regarded as cofirst authors.

## Funding

This study was supported by the National Natural Science Foundation of China (82174342, 82374382); the Joint Funds of the National Natural Science Foundation of China (U24A20778); the National Natural Science Foundation of China Youth Science Foundation Project (82505261, 82204821); the Beijing Natural Science Foundation Proposed Program (7244487); the Key Scientific Research Project of Colleges and Universities in Henan Province (24A360023); the Special Scientific Research Project on Traditional Chinese Medicine of Henan Province (2024ZY2141); the Joint Research Project of the Chinese Association of Traditional Chinese Medicine (2023DYPLHGG‐03); the High‐Quality TCM Hospitals Development Program (DZMG‐XZYY‐23002); and the Leading Talents Program of Dongzhimen Hospital, Beijing University of Chinese Medicine (DZMG‐LJRC0012).

## Ethics Statement

The experimental protocol was approved by the Animal Protection and Ethics Committee of Dongzhimen Hospital, Beijing University of Chinese Medicine (BUCM‐2023042003‐2110).

## Conflicts of Interest

The authors declare no conflicts of interest.

## Data Availability

The data that support the findings of this study are available from the corresponding authors upon reasonable request.

## References

[1] International Diabetes Federation , IDF Diabetes Atlas, 2025, 11th edition, International Diabetes Federation (IDF), http://diabetesatlas.org/.

[2] de Zeeuw D. , The Future of Diabetic Kidney Disease Management: Reducing the Unmet Need, Journal of Nephrology. (2020) 33, no. 6, 1163–1169, 10.1007/s40620-020-00820-2, 32749580. 32749580 PMC7701076

[3] Luo X. , Xu J. , Zhou S. , Xue C. , Chen Z. , and Mao Z. , Influence of SGLT2i and RAASi and Their Combination on Risk of Hyperkalemia in DKD: A Network Meta-Analysis, Clinical Journal of the American Society of Nephrology : CJASN. (2023) 18, no. 8, 1019–1030, 10.2215/cjn.0000000000000205, 37256921. 37256921 PMC10564376

[4] Kopel J. , Pena-Hernandez C. , and Nugent K. , Evolving Spectrum of Diabetic Nephropathy, World Journal of Diabetes. (2019) 10, no. 5, 269–279, 10.4239/wjd.v10.i5.269, 31139314. 31139314 PMC6522757

[5] Koch E. A. T. , Nakhoul R. , Nakhoul F. , and Nakhoul N. , Autophagy in Diabetic Nephropathy: A Review, International Urology and Nephrology. (2020) 52, no. 9, 1705–1712, 10.1007/s11255-020-02545-4. 32661628

[6] Di Malta C. , Cinqueand L. , and Settembre C. , Transcriptional Regulation of Autophagy: Mechanisms and Diseases, Frontiers in Cell and Developmental Biology. (2019) 7, 10.3389/fcell.2019.00114, 31312633. PMC6614182 31312633

[7] Settembre C. , Di Malta C. , Polito V. A. , Arencibia M. G. , Vetrini F. , Erdin S. , Erdin S. U. , Huynh T. , Medina D. , Colella P. , and Sardiello M. , TFEB Links Autophagy to Lysosomal Biogenesis, Science. (2011) 332, no. 6036, 1429–1433, 10.1126/science.1204592. 21617040 PMC3638014

[8] Zhao E. and Czaja M. J. , Transcription Factor EB: A Central Regulator of Both the Autophagosome and Lysosome, Hepatology. (2012) 55, no. 5, 1632–1634, 10.1002/hep.25619, 22517549. 22517549 PMC3707298

[9] Zhang Y. , Wu Z. , Huang Z. , Liu Y. , Chen X. , Zhao X. , He H. , and Deng Y. , GSK-3*β* Inhibition Elicits a Neuroprotection by Restoring Lysosomal Dysfunction in Neurons Via Facilitation of TFEB Nuclear Translocation After Ischemic Stroke, Brain Research. (2022) 1778, 147768, 10.1016/j.brainres.2021.147768, 34968440. 34968440

[10] Chen H. Y. , Pan H. C. , Chen Y. C. , Chen Y. C. , Lin Y. H. , Yang S. H. , Chen J. L. , and Wu H. T. , Traditional Chinese Medicine Use Is Associated With Lower End-Stage Renal Disease and Mortality Rates Among Patients With Diabetic Nephropathy: A Population-Based Cohort Study, BMC complementary and Alternative Medicine. (2019) 19, no. 1, 10.1186/s12906-019-2491-y, 30943956. PMC6448220 30943956

[11] Liu X.-J. , Hu X.-K. , Yang H. , Gui L. M. , Cai Z. X. , Qi M. S. , and Dai C. M. , A Review of Traditional Chinese Medicine on Treatment of Diabetic Nephropathy and the Involved Mechanisms, American Journal of Chinese Medicine. (2022) 50, no. 07, 1739–1779, 10.1142/S0192415X22500744. 36222120

[12] Zhang Y. F. , Zhang S. J. , and Zhang Z. Y. , Effect and Mechanism of Yiqi Yangyin Qinre Decoction on Diabetic Nephropathy Mice, Journal of Liaoning University of TCM. (2024) 26, no. 7, 16–20, 10.13194/j.issn.1673-842x.2024.07.004.

[13] Cloughand E. and Barrett T. , The Gene Expression Omnibus Database, Statistical Genomics: Methods and Protocols, 2016, Springer, 93–110, 10.1007/978-1-4939-3578-9_5, 27008011. PMC4944384 27008011

[14] Zhang L. , Shi X. , Huang Z. , Mao J. , Mei W. , Ding L. , Zhang L. , Xing R. , and Wang P. , Network Pharmacology Approach to Uncover the Mechanism Governing the Effect of Radix Achyranthis Bidentatae on Osteoarthritis, BMC Complementary Medicine and Therapies. (2020) 20, no. 1, 10.1186/s12906-020-02909-4, 32316966. PMC7171799 32316966

[15] Zhang S. , Zhang S. , Bai X. , Wang Y. , Liu Y. , and Liu W. , Thonningianin A Ameliorated Renal Interstitial Fibrosis in Diabetic Nephropathy Mice by Modulating Gut Microbiota Dysbiosis and Repressing Inflammation, Frontiers in Pharmacology. (2024) 15, 1389654, 10.3389/fphar.2024.1389654, 39193336. 39193336 PMC11347433

[16] Reagan-Shaw S. , Nihaland M. , and Ahmad N. , Dose Translation From Animal to Human Studies Revisited, FASEB Journal: Official Publication of the Federation of American Societies for Experimental Biology. (2008) 22, no. 3, 659–661, 10.1096/fj.07-9574LSF, 17942826. 17942826

[17] Zhang S. J. , Zhang S. X. , and Bai X. H. , To Investigate the Protective Mechanism of Yiqi Yangyin Qingre Decotion on Podocytes in Diabetic Nephropathy Based on the Effect of Pyroptosis and Autophagy on Inflammation, Chinese Journal of Integrated Traditional and Western Nephrology. (2025) 26, no. 5, 384–389, 10.3969/j.issn.1009-587X.2025.05.004.

[18] Chen M. M. , Jia J. H. , Tan Y. J. , Ren Y. S. , Lv J. L. , Chu T. , Cao X. Y. , Ma R. , Li D. F. , Zheng Q. S. , Liu Z. , and Li J. , Shen-Qi-Jiang-Tang Granule Ameliorates Diabetic Nephropathy via Modulating Tumor Necrosis Factor Signaling Pathway, Journal of Ethnopharmacology. (2023) 303, 116031, 10.1016/j.jep.2022.116031, 36503032. 36503032

[19] Fu Z. , Su X. , Zhou Q. , Feng H. , Ding R. , and Ye H. , Protective Effects and Possible Mechanisms of Catalpol Against Diabetic Nephropathy in Animal Models: A Systematic Review and Meta-Analysis, Frontiers in Pharmacology. (2023) 14, 1192694, 10.3389/fphar.2023.1192694, 37621314. 37621314 PMC10446169

[20] Wang N. , Feng H. , Zhang Z. , Tian H. , Gu L. , Bian Y. , and Xue M. , Danggui Buxue Decoction Regulates Autophagy to Improve Renal Fibrosis in Diabetes Through miR-27a/PI3K/AKT Pathway, Journal of Ethnopharmacology. (2025) 341, 119357, 10.1016/j.jep.2025.119357, 39800243. 39800243

[21] Li Y. , Xu M. , Ding X. , Yan C. , Song Z. , Chen L. , Huang X. , Wang X. , Jian Y. , Tang G. , Tang C. , di Y. , Mu S. , Liu X. , Liu K. , Li T. , Wang Y. , Miao L. , Guo W. , Hao X. , and Yang C. , Protein Kinase C Controls Lysosome Biogenesis Independently of mTORC1, Nature Cell Biology. (2016) 18, no. 10, 1065–1077, 10.1038/ncb3407, 27617930. 27617930

[22] Barral D. C. , Staiano L. , Guimas Almeida C. , Cutler D. F. , Eden E. R. , Futter C. E. , Galione A. , Marques A. R. A. , Medina D. L. , Napolitano G. , Settembre C. , Vieira O. V. , Aerts J. M. F. G. , Atakpa-Adaji P. , Bruno G. , Capuozzo A. , de Leonibus E. , di Malta C. , Escrevente C. , Esposito A. , Grumati P. , Hall M. J. , Teodoro R. O. , Lopes S. S. , Luzio J. P. , Monfregola J. , Montefusco S. , Platt F. M. , Polishchuck R. , de Risi M. , Sambri I. , Soldati C. , and Seabra M. C. , Current Methods to Analyze Lysosome Morphology, Positioning, Motility and Function, Traffic. (2022) 23, no. 5, 238–269, 10.1111/tra.12839, 35343629. 35343629 PMC9323414

[23] Zhou M. , Zhang S. , Bai X. , Cai Y. , Zhang Z. , Zhang P. , Xue C. , Zheng H. , Sun Q. , Han D. , Lou L. , Wang Y. , and Liu W. , Acteoside Delays the Fibrosis Process of Diabetic Nephropathy by Anti-Oxidation and Regulating the Autophagy-Lysosome Pathway, Journal of Pharmacology. (2024) 978, 176715, 10.1016/j.ejphar.2024.176715, 38852699. 38852699

[24] Nanayakkara R. , Gurung R. , Rodgers S. J. , Eramo M. J. , Ramm G. , Mitchell C. A. , and McGrath M. J. , Autophagic Lysosome Reformation in Health and Disease, Autophagy. (2023) 19, no. 5, 1378–1395, 10.1080/15548627.2022.2128019, 36409033. 36409033 PMC10240999

[25] Sakellariou D. , Tiberti M. , Kleiber T. H. , Blazquez L. , López A. R. , Abildgaard M. H. , Lubas M. , Bartek J. , Papaleo E. , and Frankel L. B. , eIF4A3 Regulates the TFEB-Mediated Transcriptional Response via GSK3B to Control Autophagy, Cell Death & Differentiation. (2021) 28, no. 12, 3344–3356, 10.1038/s41418-021-00822-y, 34158631. 34158631 PMC8630043

[26] Gu J. , Huang W. , Zhang W. , Zhao T. , Gao C. , Gan W. , Rao M. , Chen Q. , Guo M. , Xu Y. , and Xu Y. H. , Sodium Butyrate Alleviates High-Glucose-Induced Renal Glomerular Endothelial Cells Damage via Inhibiting Pyroptosis, International Immunopharmacology. (2019) 75, 105832, 10.1016/j.intimp.2019.105832, 31473434. 31473434

[27] Wang S. , Wang H. , Feng C. , Li C. , Li Z. , He J. , and Tu C. , The Regulatory Role and Therapeutic Application of Pyroptosis in Musculoskeletal Diseases, Cell Death Discovery. (2022) 8, no. 1, 10.1038/s41420-022-01282-0, 36522335. PMC9755533 36522335

[28] Samsu N. , Diabetic Nephropathy: Challenges in Pathogenesis, Diagnosis, and Treatment, BioMed Research International. (2021) 2021, 1497449, 10.1155/2021/1497449, 34307650. 34307650 PMC8285185

[29] Yaribeygi H. , Katsiki N. , Butler A. E. , and Sahebkar A. , Effects of Antidiabetic Drugs on NLRP3 Inflammasome Activity, With a Focus on Diabetic Kidneys, Drug Discovery Today. (2019) 24, no. 1, 256–262, 10.1016/j.drudis.2018.08.005, 30086405. 30086405

